# Biological Apexogenesis of Undeveloped Tooth in a Patient with Spondyloepiphyseal Dysplasia: A Case Report

**Published:** 2010-05-20

**Authors:** Hengameh Ashraf, Mahsa Eskandarinezhad

**Affiliations:** 1. Department of Endodontics, Iranian Center for Endodontic Research, Dental School, Shahid Beheshti University of Medical Sciences, Tehran, Iran.; 2. Department of Endodontics, Dental School, Shahid Beheshti University of Medical Sciences, Tehran, Iran.

**Keywords:** Apexification, Dentinal Walls, Immature, Open Apex, Permanent Tooth Regeneration

## Abstract

This case report describes treatment of a necrotic immature permanent mandibular first molar with pulpal necrosis in 9-year old female with spondyloepiphyseal dysplasia. The coronal half of the root canal was debrided with a file #30 to remove necrotic tissue, and irrigated with chlorhexidine 0.12%. Bleeding was evoked to form an intracanal blood clot; the wound was then dressed with calcium hydroxide medication and provisionally restored with GIC. This was repeated at intervals of 1, 3 and 6 months. After six months, radiographic evidence revealed thickening of dentinal walls and apical closure. The progressive increase in dentinal wall thickness and apical development suggests that desirable biologic responses can occur with this form of treatment for the necrotic open apex of immature permanent teeth.

## INTRODUCTION

Pulp necrosis of an immature tooth consequent to caries or trauma can arrest the closure of the root apices and leave the tooth with thin root canal walls that are susceptible to fracture [1]. The absence of an apical constriction makes routine root canal treatment impossible; this is because sealing open apices with conventional obturation methods is very difficult [2]. In these cases, the conventional endodontic management would be to induce apexification with Ca(OH)_2_ or encourage an apical barrier with materials such as mineral trioxide aggregate (MTA) [3][4].

However, apexification with Ca(OH)_2_ has several disadvantages including long duration and increased risk of tooth fracture because of undeveloped root canal walls [5][6]. Treatment with MTA has similar disadvantages; the tooth is left with thin dentinal walls and open apices [4]. Naturally, the ideal treatment of immature teeth with undeveloped roots would be to stimulate the regeneration of a functional pulp-dentin complex [7][8]. Revascularization of immature teeth depends mainly on: a) disinfection of the canal; b) placement of a matrix in canal for tissue in-growth c) coronal bacterial tight seal [7]. Human case series [9] and animal studies [10] have showed radiographic and histologic evidences of successful regeneration after trauma in the immature permanent teeth. There is a growing body of evidence that reports pulp regeneration in the non-vital immature permanent tooth [7][8]. The fact that regeneration of the pulp belongs to pulp-dentin complex or periodontium was unknown until now. Once the regeneration procedure is initiated, the roots of these teeth develop with the radiographic evidence of apical closure and thickening root walls [11]. One of the major factors that determine the success of this process is the disinfection of the root canal system [10][12].

Jung et al. used ciprofloxacin, metronidazole and minocycline and reported successful pulpal regeneration after disinfection in necrotic teeth [13].

Ding et al. also used the same tri-antibiotic mix and observed root development in the immature teeth with apical periodontitis [14]; another study used Ca(OH)_2_ for disinfection of the canal and observed thickening of dentinal walls and subsequent apical root development [15].

## CASE REPORT

A 9-year old girl was referred to the endodontic department of Shahid Beheshti Dental School. The patient was accompanied by her mother. Her medical history revealed spondylo-epiphyseal dysplasia disease and her general appearance was typical of this syndrome.

Spondylometaphyseal dysplasias are a group of hereditary skeletal diseases which lead to small stature. They differ in pathophysiology, heredity and in their clinical and radiographic appearance and usually require growth hormones [16]. The patient had presented with severe tooth pain in the Pedodontic Department. Pulpotomy treatment was performed and the patient was then referred to the Endodontic Department.

**Figure 1:**
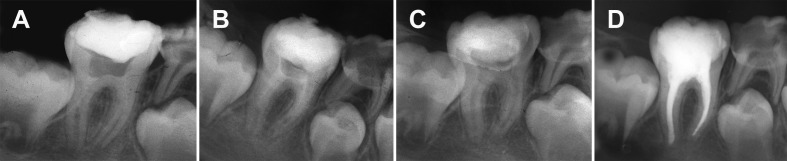
A) Periapical radiograph of first appointment, B) Periapical radiograph 3 months after first appointment, C) Periapical radiograph 6 months after first appointment, and D) Periapical radiograph of final obturation 9 months after first appointment

The patient first mandibular molar tooth was slightly symptomatic to percussion but did not respond to cold and electric pulp testing. Periodontal probing was within normal limits. Periradicular radiographic examination revealed that the roots of the right first mandibular tooth had open apices (blunderbuss mesial root canal and wide and parallel distal root canal). There was a small apical radiolucency under the mesial roots (Figure 1A). The tooth (#30) was diagnosed with a necrotic pulp consequent to caries.

Low patient cooperation i.e. limitation of mouth opening for more than 5 minutes meant rubber dam could not be applied; however isolation was achieved with high vacuum suction, gauze and cotton rolls.

At the first visit, patient received local anesthesia with lidocaine with 1:100,000 epinephrine. The access cavity was made and the canal mechanically prepared for approximately 1/3 of roots length by using K-file #40. Root canals were irrigated with chlorhexidine 0.12% (Irsha factory), and dried with paper points. Calcium hydroxide powder medication was mixed with saline to achieve a pasty consistency and was placed into the canal by using file #40. The access cavity was sealed with glass ionomer as the temporary restoration.

At the 1 month follow-up visit, the tooth was asymptomatic and not sensitive to percussion. Ca(OH)_2_ dressing was removed by rising with chlorhexidine 0.12%, the canal was dried with paper points, a K-file #30 was inserted into the canal. The patient did not complain of any sensation until the file tip reached approximately the middle part of the canal. At this point, patient reported pain and haemorrhage was observed from the canal. Thus, it was concluded that some vital pulp tissue was likely to be present within the canal which made pulp regeneration possible. After formation of blood clot the Ca(OH)_2_ powder dressing was placed over the clot and tooth was restored with glass ionomer.

Three months post-operatively, follow up radiographic revealed progressive development of roots (Figure 1B). The tooth was also asymptomatic. Calcium hydroxide dressing was placed as before.

At the six-month follow up, radiographic evaluation of the tooth showed apical root apices closure, increased root canal walls thickness and periapical health (Figure 1C).

After removal of the Ca(OH)_2_, existence of barriers was confirmed with radiographs and files. Routine obturation with gutta-percha was performed followed by a bonded resin restoration (Figure 1D).

The one-year follow up revealed no signs or symptoms; also, closure of the apices and thickening of dentinal walls were observed.

## DISCUSSION

The traditional treatment for necrotic immature permanent teeth is the complete pulpectomy of the tooth and apexification with calcium hydroxide or MTA. However, this method does not induce further thickening of dentinal walls [3][17], and therefore the risk of root fracture remains and better treatment alternatives become desirable [1].

Remaining vital pulp tissue in teeth with open apices could regenerate and induce morpho-differentiation of stem cells from the dental papilla or apical periodontium; therefore the elimination of bacteria from the pulp is of paramount importance to allow successful regeneration [10][12].

In this case study the full length of the root canals was not cleaned as the patient experienced pain when the file was inserted fully. This suggests the presence of apical residual vital pulp tissue within the canals. Therefore the canals were disinfected up to the remaining vital pulp tissue and then irrigated with chlorhexidine. A file #30 was used to create a blood clot, which acts as scaffold for growing tissue [18][19]. In this study Ca(OH)_2_ dressing was used due to its antimicrobial effect, clinical availability and cost effectiveness (compared to MTA). After 6 months, first visit radiographic examination revealed apical closure and an increase of thickness of the canal walls with reduction of pulp space. In the clinical examination canals were reopened and a hard tissue barrier at the apices was felt by using a file #40.

Cotti et al. [15] used Ca(OH)_2_ powder as an intracanal medication similar to our study, however, disinfection and removed of Ca(OH)_2_ was performed with hypochlorite sodium 5.25% at the following appointment and after drying the canal with paper points, formation of blood clot was stimulated. MTA was used as the final dressing over the clot. The access cavity was sealed with cavit. The 8 months follow up showed apical closure. They concluded that stem cells originating from pulp or periodontium could induce the continued regeneration of the root. Patient in our study used growth hormone; it is possible that the growth hormone could affect development of root in a positive way.

Jung et al. used ciprofloxacin, metronidazole, and minocycline as an intracanal medication. After 11 days, the root canal was flushed with NaOCl 5.25% and dressed with MTA [13]. Their method achieved apexogenesis after 3 months.

A further study also applied metronidazole and minocycline into the root canal system; after 26 days, they created a blood clot with an explorer [7] and then placed MTA over the pulp wound. The 2-year follow up revealed closure of the apex and thickening of dentinal walls.

Similar results were observed in a study which used NaOCl 2.5% irrigation and Ca(OH)_2_ medication [8]. In these cases developing apices with thickening dentinal walls was observed after 7 months.

In these successful cases the composition of the dentinal wall, i.e. dentin or cementum, was not established. The cells may have originated from the periodontal ligament that had grown into the canal space to deposit cementum unto the inner surface of the root dentin [10][20]. They could also have originated from the remaining vital pulp tissue; as stem cells in the apical papilla have the ability to proliferate and form odontoblast-like cells [21][22]. Moreover, the regular intake of growth hormone may also have been an influential factor: tissue regeneration is not a common consequence in canal treatment with Ca(OH)_2_.

## CONCLUSION

Despite the low success rate of this form of treatment, the present case demonstrated that the regeneration of the pulp of immature teeth with partial necrosis is possible and this treatment should be preferred to the conventional apexification treatment.

Further research in this field is necessary to support these recommendations.
